# Glucose-6-Phosphatase Catalytic Subunit 3 (*G6PC3*) Deficiency Associated With Autoinflammatory Complications

**DOI:** 10.3389/fimmu.2017.01485

**Published:** 2017-11-06

**Authors:** Anoop Mistry, Thomas Scambler, David Parry, Mark Wood, Gabriela Barcenas-Morales, Clive Carter, Rainer Doffinger, Sinisa Savic

**Affiliations:** ^1^Department of Clinical Immunology and Allergy, St James’s University Hospital, Leeds, United Kingdom; ^2^National Institute for Health Research–Leeds Biomedical Research Centre (NIHR–LMBRU), Leeds Institute of Rheumatic and Musculoskeletal Medicine (LIRMM), St James’s University Hospital, Leeds, United Kingdom; ^3^Centre for Genomic and Experimental Medicine, Institute of Genetics and Molecular Medicine, University of Edinburgh, Edinburgh, United Kingdom; ^4^Department of Paediatrics Rheumatology, Leeds General Infirmary, Leeds, United Kingdom; ^5^Laboratorio de Inmunologia, FES-Cuautitlan, Universidad Nacional Autónoma de México, Mexico City, Mexico; ^6^Department of Clinical Biochemistry and Immunology, Addenbrooke’s Hospital, Cambridge, United Kingdom

**Keywords:** *G6PC3*, systemic autoinflammatory syndromes, PID, neutropenia, adalimumab

## Abstract

*G6PC3* deficiency typically causes severe congenital neutropenia, associated with susceptibility to infections, cardiac and urogenital abnormalities. However, here we describe two boys of Pakistani origin who were found to have G6PC3 deficiency due to c.130 C>T mutation, but who have clinical phenotypes that are typical for a systemic autoinflammatory syndrome. The index case presented with combination of unexplained fevers, severe mucosal ulcers, abdominal symptoms, and inflammatory arthritis. He eventually fully responded to anti-TNF therapy. In this study, we show that compared with healthy controls, neutrophils and monocytes from patients have reduced glycolytic reserve. Considering that healthy myeloid cells have been shown to switch their metabolic pathways to glycolysis in response to inflammatory cues, we studied what impact this might have on production of the inflammatory cytokines. We have demonstrated that patients’ monocytes, in response to lipopolysaccharide, show significantly increased production of IL-1β and IL-18, which is NLRP3 inflammasome dependent. Furthermore, additional whole blood assays have also shown an enhanced production of IL-6 and TNF from the patients’ cells. These cases provide further proof that autoinflammatory complications are also seen within the spectrum of primary immune deficiencies, and resulting from a wider dysregulation of the immune responses.

## Background

Systemic autoinflammatory disorders (SAIDs) can manifest as periodic sterile fevers, with or without tissue specific signs and symptoms such as skin rashes, serositis, synovitis, and mucosal ulceration. In well-defined monogenic SAIDs, the pathogenic mutation typically leads to gain-of-function within the innate-immune-mediated inflammatory pathways (1). However, it is increasingly recognized that autoinflammatory complications are also seen within the spectrum of primary immune deficiencies (2). Here, we describe the case of two brothers who presented with SAID but were found to have a mutation in *G6PC3*. This mutation typically causes cardiac and urogenital abnormalities (3–6), and severe congenital neutropenia associated with susceptibility to infections, but not usually SAID.

## Case Presentation

The index patient is a 12-year-old male, who was born healthy at term to consanguineous parents of Pakistani origin. At 1 year of age, he developed mild, self-limiting oral aphthous ulcers. Neutropenia was first noted on an incidental blood test when he was 3 years old. From the age of 7 years, he suffered progressive, deep oral ulceration severe enough to result in complete dysphagia and several hospital admissions. He also developed transient perianal ulceration associated with episodic diarrhea and intermittent abdominal pain. Other pertinent features included a swinging pyrexia (39°C), oligoarthritis (knees) associated with a joint effusion on at least one occasion, and cervical lymphadenopathy. At the age of 11, he developed a rash on his right shin. The rash was diagnosed as erythema nodosum. He had no eye symptoms, or any other organ involvement. The only notable infections were paronychial nail infections and a conjunctival infection due to herpes simplex virus. The patient had two other siblings, and only the younger brother who initially was asymptomatic, later developed similar but less severe clinical features. There was no other significant family history of inflammatory disorders. The index patient responded well to prednisolone, but he remained dependent on high doses of the steroid. The trial of colchicine was ineffective, but he subsequently responded fully to adalimumab.

## Clinical and Laboratory Investigations

**Figure 1:**
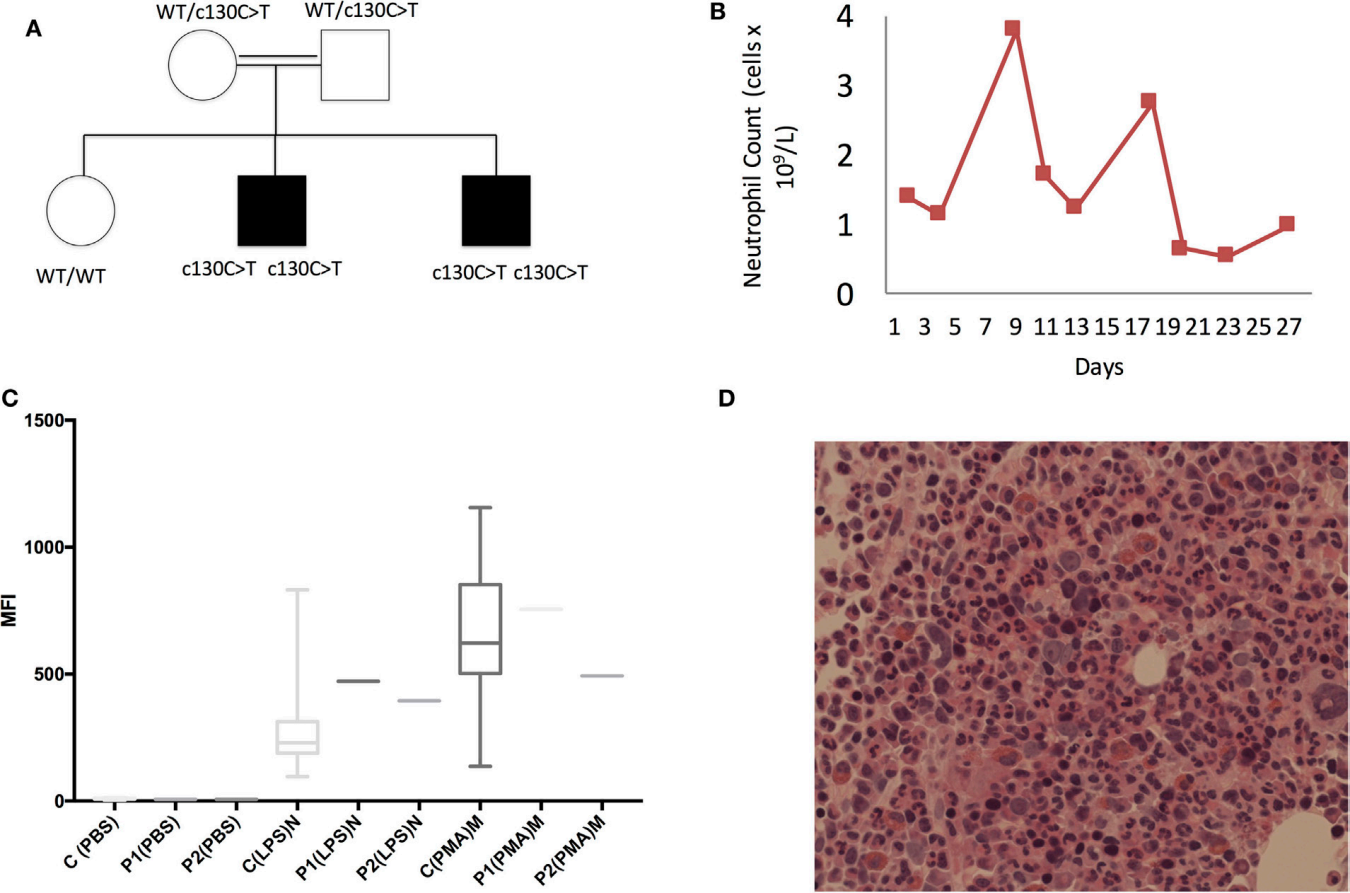
Clinical characteristics of patients with *G6PC3* c.130 C>T homozygous mutation. **(A)** Family pedigree, **(B)** longitudinal neutrophil count measurements, **(C)** neutrophil oxidative burst. Local reference ranges are shown in the bars. >98% of neutrophils and monocytes showed appropriate shift following lipopolysaccharide (LPS) and phorbol myristate acetate (PMA) stimulation. **(D)** Histology slide from bone marrow biopsy showing normal neutrophil maturation.

Routine investigations revealed a cyclical neutropenia (Figure 1B) and negative autoimmune screen, which included antinuclear and antineutrophil cytoplasmic antibodies. Septic screen was also negative and included chest X-ray, blood cultures, and PCR for Epstein–Barr virus, cytomegalovirus, and adenovirus. During flares, he was found to have increased acute phase response with highly elevated C-reactive protein (300 mg/L), elevated serum amyloid A (45.2 mg/L), normocytic anemia, and polyclonal increase in IgG and IgM. Extensive investigations of the gastrointestinal tract included fecal calprotectin, which was raised at 600 µg/g (normally <50 μg/g), normal abdominal ultrasound, endoscopy, and colonoscopy with biopsies taken from the mouth ulcers, esophagus, antrum, duodenum, ileum, and throughout the large colon. Endoscopy showed generalized inflammation from distal esophagus to duodenum, while colonoscopy revealed localized areas of edema and ulceration in terminal ileum. The biopsies of the mouth ulcers showed marked diffuse chronic inflammation with an infiltrate of lymphocytes and plasma cells as well as focal superficial acute inflammation with ulceration of the mucosa and acute purulent exudate. There were no well-formed discrete granulomas seen. Biopsies from the esophagus, antrum, duodenum, and ileum were all within normal limits. The colonic biopsies showed an equivocal mild increase in chronic inflammatory cells in the lamina propria. There was no evidence of active inflammation, glandular/architectural distortion, or granulomata. These changes were thought to be mild and of questionable significance. In summary, there was no definite evidence on these biopsies to support a formal diagnosis of inflammatory bowel disease (IBD). A bone marrow biopsy showed no obvious defects in neutrophil production or maturation (Figure 1D). Plain X-rays of the hips, knees, and pelvis taken during one of the flare episodes were all normal. An ultrasound scan of the hips and knees, which was done at the same time, did not show any effusion or synovial thickening. Subsequent to these investigations the patient did develop a clinically evident left knee effusion. Analysis of the synovial fluid during this episode showed heavy presence of polymorphs and mononuclear cells, with no visible crystals. Gram stains were negative, and subsequent synovial fluid culture was sterile.

Diagnoses of SAID and Behcet’s disease (BD) were considered. The genetic tests limited to analysis of *MEFV, MVK*, and *TNFRSF1A* genes were negative, but he was found to be HLA-B51 positive. However, since several clinical features were not typical of BD the family was consented for whole-exome sequencing. Both brothers were found to have a homozygous mutation in *G6PC3* c.130 C>T, p.(Pro44Ser) (Figure 1A).

**Figure 2:**
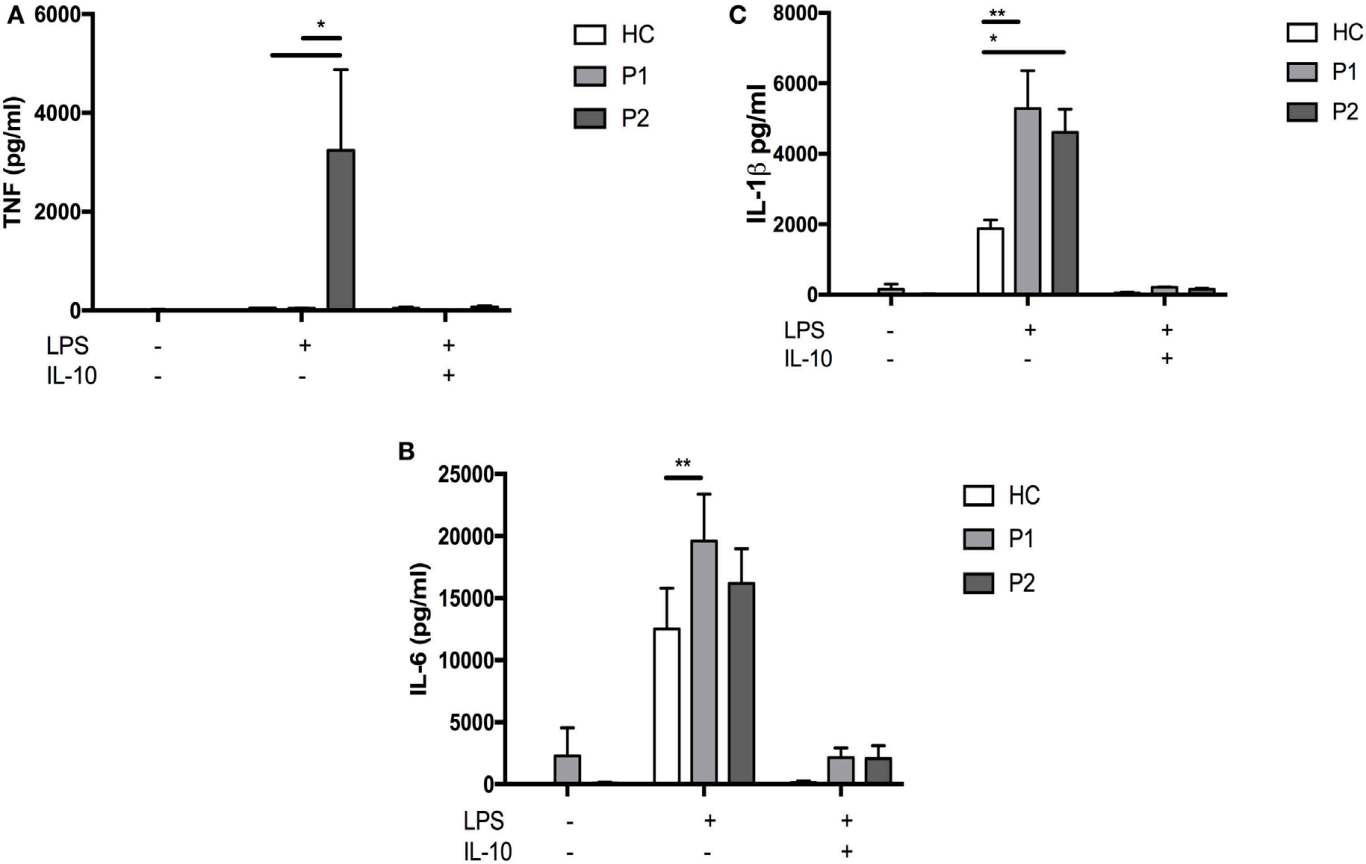
Whole blood lipopolysaccharide (LPS) stimulation and cytokine responses. Whole blood was diluted 1:5 with Roswell Park Memorial Institute medium into 96-well F plates (Corning) and activated by single stimulation or co-stimulations as indicated with LPS (1 µg/mL; List Biochemicals), + others. Supernatants were taken at 24 h. Cytokines were measured with multiplexed particle based flow cytometry **(A)** TNFa **(B)** IL6 **(C)** IL-1β; (All measured with R+D Systems Fluorokinemap) on a Luminex analyzer (Bio-Plex, Bio-Rad, UK). Two-way ANOVA statistical test was used (*p* < 0.05).

Further immunological assessment showed that brothers had a normal neutrophil oxidative burst in response to lipopolysaccharide (LPS) and phorbol myristate acetate stimulation (Figure 1C). Since their clinical phenotypes were in keeping with SAID, we tested their ability to produce pro-inflammatory cytokines *in vitro*. The assays performed using the whole blood confirmed that the patients’ cells produced significantly higher levels of IL-1β and IL-6 compared with healthy control (HC) following 24-h stimulation with LPS (Figures 2B,C). The younger brother also showed increased TNF production (Figure 2A); at the time of testing, the older sibling was receiving anti-TNF therapy (adalimumab). The addition of IL-10 to the whole blood assay attenuated increased production of IL-1β, IL-6, and TNF, however, in the case of IL-1β, this was reduced in both brothers compared with HC (Figure 2).

**Figure 3:**
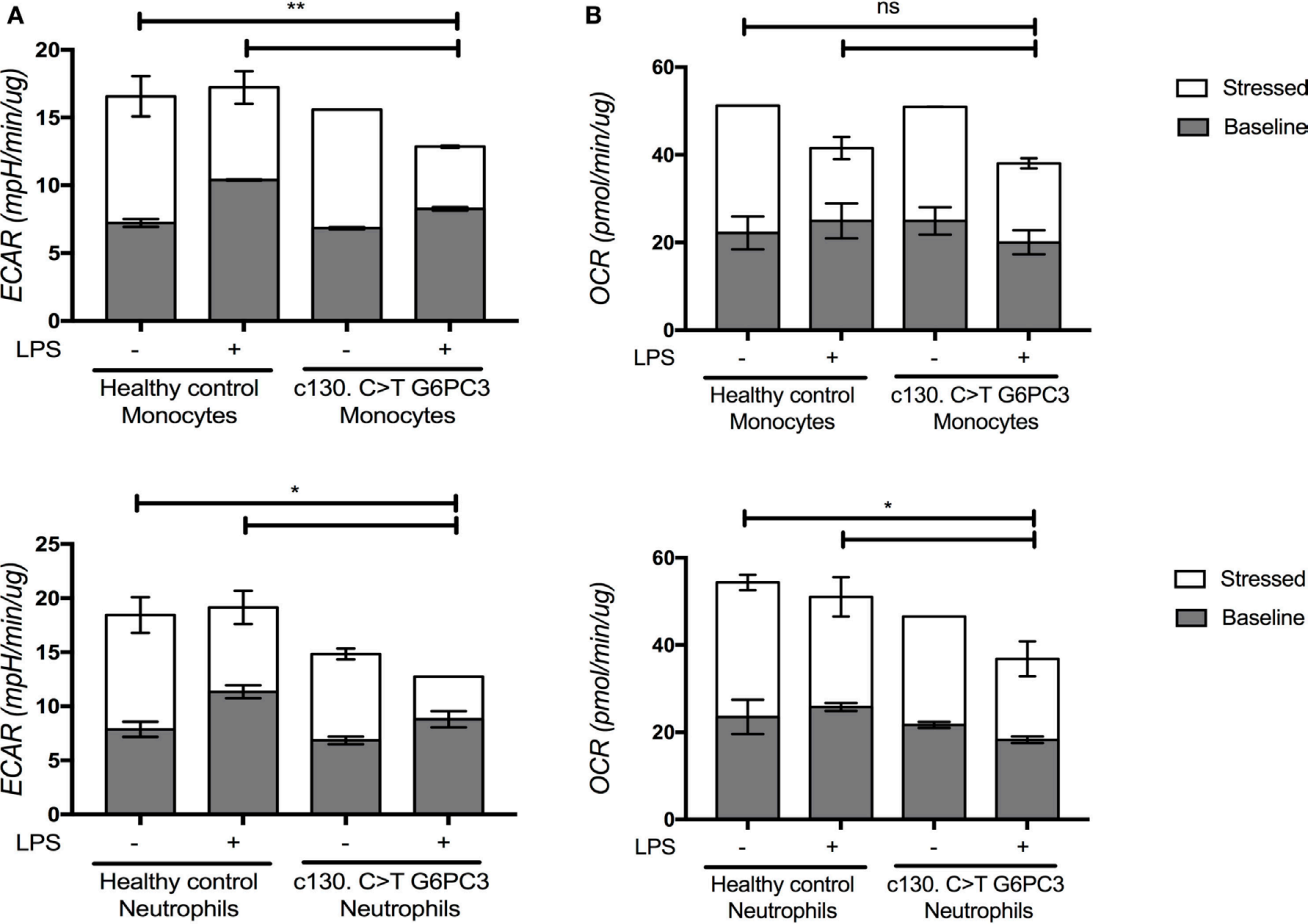
Levels of glycolysis and oxidative phosphorylation within monocytes and neutrophils from patients and healthy controls under basal and stressed conditions, with and without lipopolysaccharide (LPS) stimulation. Neutrophils were separated from the erythrocyte/granulocyte layer using ammonium chloride red cell lysis buffer (Sigma) after separating PBMC from the whole blood using the lymphoprep technique. Monocytes were separated further using magnetic beads (Miltenyi Biotec) for monocyte negative selection. Granulocytes and monocytes were seeded into the Agilent 96-well flux plate at 5 × 10^5^ cells/well in triplicate wells and stimulated with or without LPS (10 ng/mL) (Invivogen) for 4 h. The XFp Cell Energy Phenotype Test (Agilent) was performed using the Agilent Xfe Seahorse Extracellular Flux Analyzer. Extracellular acidification rate (ECAR) and oxygen consumption rate (OCR) were measured by the Agilent Seahorse Metabolic Profiler using the Basal Metabolic Phenotype Assay as an indirect measurement of glycolysis and oxidative phosphorylation (OXPHOS) within live cell cultures. The baseline levels (gray) represent glycolysis **(A)** or OXPHOS **(B)** within the cells before metabolic stressors. The stressed levels (superimposed bars in white) represent glycolysis or OXPHOS post-oligomycin and FCCP injection. The two-way ANOVA statistical test was used (*p* < 0.05) and refers to the stressed (white) conditions.

**Figure 4:**
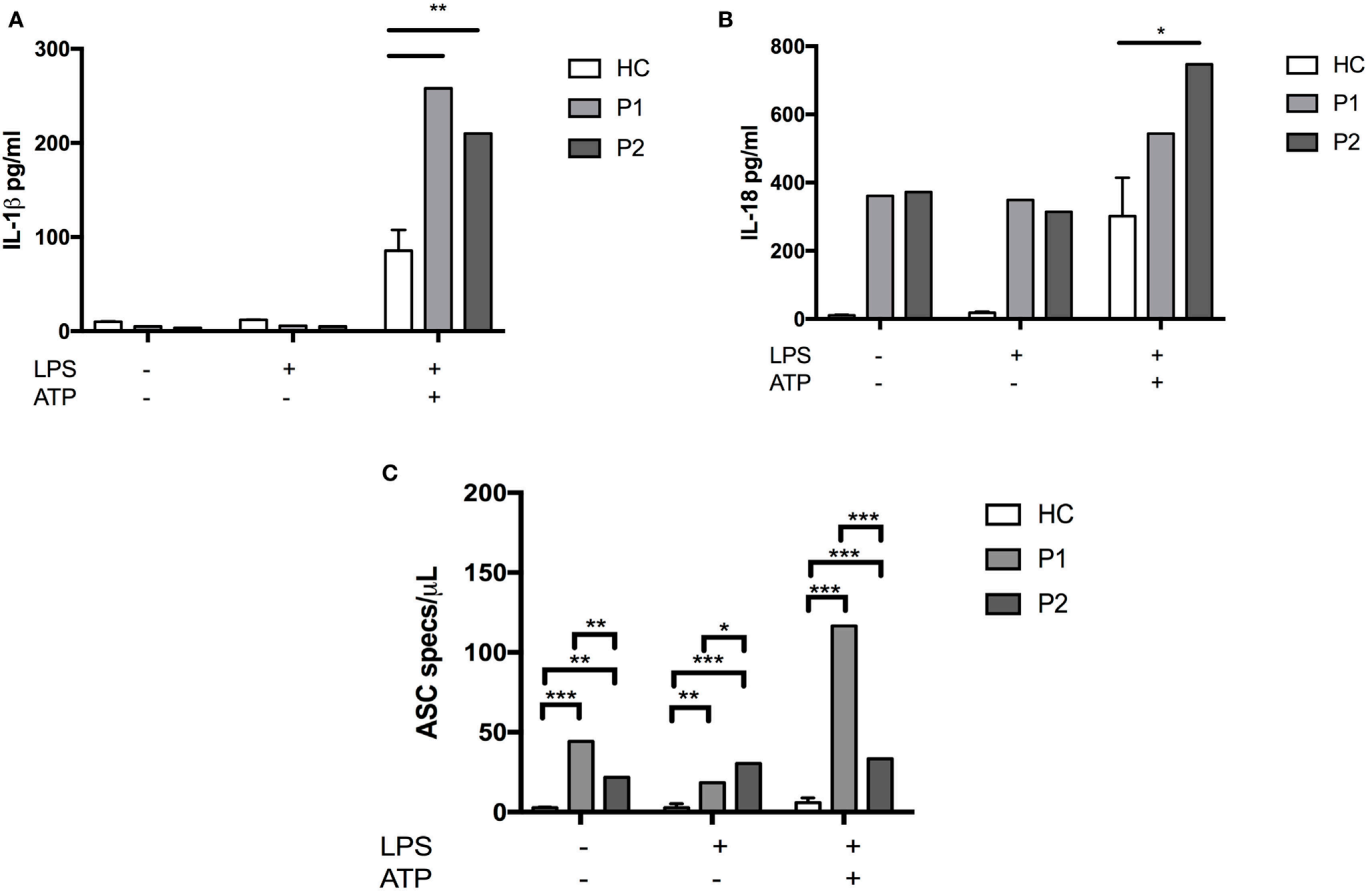
IL-1β, IL-18 levels, and apoptosis associated speck-like protein containing a CARD (ASC) particle numbers following stimulation of monocytes with lipopolysaccharide (LPS)/ATP. Patients’ and healthy control (HC) monocytes were seeded at 1 × 10^6^ cells/mL and stimulated with LPS (10 ng/mL) (Invivogen) for 4 h and then ATP (1 mM) (Invivogen) for 30 min. Supernatants were collected and used for IL-1β and IL-18 ELISA (ThermoFisher). The ASC particles were measured using directly conjugated anti-ASC ab (Cambridge Bioscience) and particles visualized by flow cytometry (BD LSRII). Two-way ANOVA statistical test was used (*p* < 0.05).

We then tested the ability of patients’ neutrophils and monocytes to metabolically respond to LPS, specifically to induce glycolysis under conditions of metabolic stress. Healthy myeloid cells have been shown to switch their metabolic pathways to glycolysis in response to inflammatory cues such as LPS (7, 8). Given the important role of G6PC3 in gluconeogenesis and glycolysis, we hypothesized that this metabolic reprogramming might be impaired, predisposing enhanced production of pro-inflammatory cytokines. We found that at baseline, G6PC3 monocytes and neutrophils had comparable levels of glycolysis, measured by extracellular acidification rate (Figure 3A). Following stimulation with LPS, both neutrophils and monocytes induced glycolysis as expected; however, this metabolic reprogramming was significantly reduced in G6PC3 cells, indicating insufficient utilization of glycolysis in response to LPS. However, no obvious change in mitochondrial oxidative phosphorylation (OXPHOS), measured by oxygen consumption rate, was observed (Figure 3B) with the addition of LPS in monocytes from either HC or G6PC3 cells, although G6PC3 neutrophils had significantly reduced OXPHOS at baseline. We wondered if the metabolic changes we observed in granulocytes and monocytes had a more specific effect on NACHT, LRR, and PYD domains-containing protein 3 (NLRP3) inflammasome complex, which is responsible for processing of pro-IL-1β and pro-IL-18 into their active components and is frequently dysregulated in many monogenic SAIDs. We found that the production of IL-1β and IL-18 from patients’ monocytes was significantly increased, compared with HC, after stimulation of NLRP3 by priming and activation with LPS and ATP, respectively (Figures 4A,B). This was associated with increased release of apoptosis associated speck-like protein containing a CARD (ASC) specks (Figure 4C), which are typically released after stimulation of the NLRP3 inflammasome as the activated cells die by pyroproptosis.

## Discussion

Although G6PC3 deficiency is typically associated with predisposition to recurrent severe infections and congenital/organ abnormalities, inflammatory complications have also been described. For example, IBD resembling Crohn’s disease (CD) was noted in 3/14 patients in one case series (5), and in another report such complications were identified in 5/57 patients, with two patients being a sibling pair (6). Taken altogether, these reports suggest that the incidence of IBD in patients with G6PC3 deficiency is higher compared with the general population (6). Furthermore, the clinical course of IBD in some of these cases seems to be severe, with two patients reported to have developed bowel stenosis requiring a partial colectomy (5) and another patient needing anti-TNF therapy following similar IBD-related complication (9). In at least one case, IBD was associated with a non-destructive polyarthritis, which was treated with corticosteroids (5). IBD as a complication of neutrophil-centered PID is not unique to G6PC3 deficiency, as it is also seen in other primary neutropenias (10–12) and in chronic granulomatous disease (CGD) (13). This association between inadequate neutrophil numbers or function and IBD is not entirely surprising, given that failure to adequately control gut microflora leading to dysregulated inflammatory responses in the gut, has also been linked with the pathogenesis of CD itself (14, 15).

In regard to the specific *G6PC3* mutation we describe here, there are three additional patients with the same c.130 C>T homozygous variant reported in the literature (6). They were also of Pakistani origin, with no congenital/organ abnormalities, and one patient presented with mouth ulcers and transient myositis. Although patients with c.130 C>T genotype appear to have a milder phenotype, this is not necessarily associated with preservation of protein function. *In vitro* experiments show that this mutation causes almost total loss of phosphohydrolase activity of the G6Pase-β mutant (16). Interestingly, this does not appear to significantly affect neutrophil oxidative burst activity, which is in keeping with the clinical phenotype of our patients who did not suffer from serious infections. The lack of glycolytic responsiveness to LPS may affect other neutrophils responses. We have shown increased production of inflammatory cytokines from whole blood and reduced glycolysis of patient’s neutrophils and monocytes in response to LPS. The latter was evident once the cells were put under metabolic stress due to LPS stimulation and a cocktail of oligomycin and carbonyl cyanide 4-(trifluoromethoxy)phenylhydrazone (FCCP), and was associated with increased production of mature IL-1β, IL-18, and release of ASC specs. The impaired glycolytic function might result in insufficient ATP production and a resultant energy deprivation. Others have described this hypoglycolytic phenomenon in other immunological diseases such as rheumatoid arthritis (RA), with deficiencies in the glycolytic enzyme phosphofructokinase (PFKFB3) (17). PFKFB3 deficiency and impaired glycolysis caused glucose to enter the pentose phosphate pathway in RA T cells. PFKFB3 deficiency induced energy deprivation, increased NADPH, increased ROS consumption, impaired autophagy, and increased apoptosis. Our data suggest that hypoglycolytic monocytes and neutrophils are pro-inflammatory and have increased pyroptosis, a form of inflammatory cell death associated with ASC spec release into the extracellular space. Increased neutrophil cell death is known to occur in G6PC3 deficiency (18). Consequently, an energy deprived, hypoglycolytic cell might result in alterations in redox balance and reactive oxygen species consumption, known to activate the NLRP3 inflammasome (19).

It is important to appreciate the range of autoinflammatory complications associated with PIDs, since different treatment options might need to be considered. In the case of CGD, this concept has evolved significantly (20) and biological therapies targeting inflammatory cytokines, both TNF and IL-1 have now been used successfully to treat IBD and other associated autoinflammatory complications (21, 22). Similarly in this case, anti-TNF therapy has proven to be safe and effective treatment. However, using such therapies in PID cases does carry extra risk and requires increased vigilance for signs of emerging infective complications (23).

## Concluding Remarks

In summary, we show that a rare *G6PC3* genotype can present with unique and varied clinical features in keeping with SAID. Furthermore, we show that hypoglycolytic responses and dysregulated production of pro-inflammatory cytokines might be central to this phenotype. Finally, we report that anti-TNF therapy is a safe and effective treatment for some of these patients.

## Ethics Statement

The study was performed in accordance with the Declaration of Helsinki. Human samples were collected with ethical approval from Leeds Ethics Committees, with written informed consent from participants before inclusion in the study.

## Author Contributions

AM collected clinical data and was involved in the care of the patients. TS performed seahorse and inflammasome-related experiments. DP performed genetic analysis (whole-exome sequencing). MW was the principal clinician in charge of the patients care. CC performed routine immunological investigations including NFT. RD and GB-M performed whole blood cytokine experiments. All the authors helped to write the paper. SS designed the study, collected clinical information, wrote the majority of the manuscript, and was involved in the patients’ clinical care.

## Conflict of Interest Statement

The authors declare that the research was conducted in the absence of any commercial or financial relationships that could be construed as a potential conflict of interest.

## Abbreviations

ASC, apoptosis associated speck-like protein containing a CARD; CRP, C-reactive protein; G6PC3, glucose-6-phosphatase catalytic subunit 3; IBD, inflammatory bowel disease; IL, interleukin; LPS, lipopolysaccharide; *MEFV*, Mediterranean fever; ROS, reactive oxygen species; MVK, mevalonate kinase; NLRP3, NACHT, LRR, and PYD domains-containing protein 3; PMA, phorbol myristate acetate; SAA, serum amyloid A; SAID, systemic autoinflammatory disorder; *TNFRSF1A*, tumor necrosis factor receptor superfamily member 1A.
